# Supplementary material for: Are Parent-Held Child Health Records a Valuable Health Intervention? A Systematic Review and Meta-Analysis
Source: Int J Environ Res Public Health. 2019 Jan 14;16(2):220. doi: 10.3390/ijerph16020220 (PMC6352207; doi:10.3390/ijerph16020220)
Supplement: Supplementary file 1 [file ijerph-16-00220-s001.pdf]

Table S1. List of excluded studies after full text screening.

Study		Reason for Exclusion
1.	Aihara [63]	No child outcome
2.	Kients et al. [64]	No child outcome
3.	Farida [13]	No child outcome
4.	Garg et al. [10]	No child outcome
5.	Kitabayashi et al. [65]	No child outcome
6.	Kusumayati and Nakamura [4]	No child outcome
7.	Mori et al. [5]	No child outcome
8.	Ahmadi et al. [66]	Secondary study/ Review of existing data
9.	Brown et al. [67]	Secondary study/ Review of existing data
10.	Nwacheck and Halfon, [68]	Secondary study/ Review of existing data
11.	Hooker [11]	Secondary study/ Review of existing data
12.	McElligott and Darden [8]	Secondary study/ Review of existing data
13.	Moss 2005 [69]	Secondary study/ Review of existing data
14.	Mudany et al. [3]	Secondary study/ Review of existing data
15.	Osaki et al. [70]	Secondary study/ Review of existing data
16.	Osaki et al. [71]	Secondary study/ Review of existing data
17.	Osaki et al. [72]	Secondary study/ Review of existing data
18.	Riverin et al. [12]	Secondary study/ Review of existing data
19.	Shah et al. [7]	Secondary study/ Review of existing data
20.	Takeuchi et al. [2]	Secondary study/ Review of existing data
21.	Tom et al. [73]	Secondary study/ Review of existing data
22.	Calvin [15]	No full text found
23.	Cohen et al. [14]	No full text found
24.	Mahomed et al. [74]	No full text found
25.	Volkmer et al. [1]	No full text found
26.	Kelly [75]	Participants not Parents
27.	Whetstone and Goldsmith [76]	Participants not Parents
28.	Carr et al. [77]	Not parent-held record
29.	Eapen et al. [78]	Not parent-held record
30.	Froen et al. [79]	Not parent-held record
31.	Knowles et al. [80]	Not parent-held record
32.	Thomas et al. [81]	Not parent-held record
33.	Knight et al. [82]	Not child record
34.	Kim et al. [83]	Not child record
35.	Kim et al. [84]	Not child record
36.	Reich et al. [85]	Not child record
37.	Rybynok et al. [86]	Not child record
38.	White [87]	Not child record
39.	Nurhayati et al. [88]	No full-text in English
40.	Fujimoto et al. [9]	No full-text in English
41.	McMaster et al. [6]	Children aged above 12 years included
42.	Wenzel [89]	Child age not from birth

Table S2. Data Extraction Form adapted from the Cochrane Collaboration.

<u>Title of the systematic review:</u> **Are parent-held child health records a valuable health intervention? A Systematic review and meta-analysis**

<u>Trial Registration no:</u> **CRD42018096209**

This form has been developed by adopting and customizing the "Data collection form for intervention review—RCTs and non-RCTs" of The Cochrane Collaboration. Some new sections have been added into this tool and the irrelevant sections have been removed from the original form. Information included on this form should be comprehensive, and may be used in the text of the review.

<u>Notes on using this data extraction form:</u>

Be consistent in the order and style you use to describe the information for each included study

Record any missing information as unclear or not described, to make it clear that the information was not found in the study report(s), not that you forgot to extract it.

Include any instructions and decision rules on the Data Extraction Form, or in an accompanying document. It is important to practice using the form and give training to any other authors using the form.

We will protect the document in order to use the form fields (Tools / Protect document)

**1. General Information**	
1. Date form completed *(dd/mm/yyyy)*	
2. Name/ID of person extracting data	
3. Report title *(title of paper/ abstract/ report that data are extracted from)*	
4. Report contact details of person extracting data	
5. Publication type *(e.g., full report, abstract, letter)*	
6. Study ID *(e.g., 01 plus surname of first author and year first full report of study was published e.g., Smith 2001)*	
7. Country in which the study conducted	
8. Economic level of the country in which the study conducted *(e.g., low income, lower-middle income or upper-middle income)*	
9. Study funding source *(including role of funders)*	
10. Possible conflicts of interest *(for study authors e.g., not reported)*	
11. **Notes:**	
**2. Eligibility**	

Study Characteristics	Review Inclusion Criteria *(Insert inclusion criteria for each characteristic as defined in the Protocol e.g. cross-sectional, cohort or case-control)*	**Location in text** *(page#/fig /table)*
12. Type of study		P2
13. Population description		P2
14. Focused diseases / conditions *(Urinary incontinence, Faecal incontinence, pelvic organ prolapse, or at least one of them)*		P2
15. Types of outcome measures *(Prevalence/Risk factors)*		P1<br>P1
16. Decision *(with reasons for either inclusion or exclusion)*		
17. **Notes:**		
**Do not proceed if study is excluded from review**<br>**3. Population and setting**		
	Description	Location in text *(page#/fig /table)*
18. Population description *(from which study participants are drawn)*		
	Description	Location in text *(page#/fig /table)*
19. Source/setting of the population *(e.g. urban, rural, particular ethnic group)*		
20. Method/s of recruitment of participants		
21. **Notes:**		
**4. Methods**		
	Descriptions as stated in report/paper	Location in text *(page#/fig /table)*

22. Aim of study		
23. Design *(e.g., cross-sectional study, cohort study, case-control study)*		
24. Sampling technique *(e.g. random or convenience)*		
25. Study start date		
26. Study End date/duration *(if any cohort)*		
27. **Notes:**		
**5. Participants** *Provide overall data and, if available, comparative data for each intervention or comparison group.*		
	Description as stated in report/paper	Location in text *(page#/fig /table)*
28. Total number of participants/Sample size		
29. Age group		
	Description as stated in report/paper	Location in text *(page#/fig /table)*
30. Menopause status *(if any)*		
31. **Notes:**		
**6. Outcomes**		
How outcomes measured	Description as stated in report/paper	Location in text *(page#/fig/ table)*
32. Outcomes *(detected by physical examination: who examined?)*		
33. Self-reported reported outcomes *(detected by questionnaire: validated or non-validated?)*		

Outcome 1: Prevalence<br><br>(Note: Not detail here under outcome. Detail should be reported in results section)	Description as stated in report/paper	Location in text *(page#/fig /table)*
34. Outcome names<br>*(Urinary incontinence, Faecal incontinence, pelvic organ prolapse, or at least one of them)*		
35. Time points measured *(report the start year/specify whether from start and end of intervention)*		
36. Time points reported		
Outcome 1: Prevalence<br><br>(Note: Not detail here under outcome. Detail should be reported in results section)	Description as stated in report/paper	Location in text *(page#/fig /table)*
37. Outcome definition *(e.g. whether standard case definition used: some standard definitions are: Pelvic Organ Prolapse Distress Inventory 6 (POPDI-6), Colorectal- Anal Distress Inventory 8 (CRADI-8), Question for Urinary Incontinence Diagnosis (QUID), Urinary Distress Inventory 8 (UD1-6), International Consultation on Incontinence Society (ICIS) etc.)*		
38. Type of measurement *(Percentage/Odds ratio/Risk ratio)*		
39. Is outcome/tool validated? *(Yes/No/Unclear/Not mentioned)*		
40. **Notes:**		
Outcome 2: Risk factors<br><br>(not detail here)	Description as stated in report/paper	Location in text *((page#/fig /table)*
41. Name of the risk factors (e.g. risk factors of POP)		
42. Time points measured *(report the start year/specify whether from start and end of intervention)*		
43. Time points reported		

44. Definition of risk factors (if any)		
45. Type of measurement (Percentage/Odds ratio/Risk ratio)		
46. Is outcome/tool validated? (Yes/No/Unclear/Not mentioned)		
47. **Notes:**		

**7. Results and findings** *Copy and paste the appropriate table for each outcome, including additional tables for each time point and subgroup as required.*		
Outcome 1: Prevalence<br><br>(Note: detail here)	Description as stated in report/paper	Location in text *(page#/fig /table)*
48. Outcome		
49. Subgroup *(if any, e.g., age- specific prevalence reporting)*		
50. Results		
51. Response/non-response rate		
52. Any other results reported		
53. Unit of analysis *(e.g., by individuals)*		
54. Statistical methods used and appropriateness of these methods *(e.g. proportion/%s, RR/OR)*		
55. Whether results weighted? *(e.g., Yes/No)*		
56. **Notes:**		
Outcome 2: Risk factors<br><br>(Note: detail here)	Description as stated in report/paper	Location in text *(page#/fig /table)*
57. Name of the risk factors<br>NB this is confusing; change to RF?		
58. Results		
59. Response/non-response rate		

60. Any other results reported		
61. Unit of analysis *(e.g., by individuals)*		
Outcome 2: Risk factors<br><br>(Note: detail here)	Description as stated in report/paper	Location in text *(page#/fig /table)*
62. Statistical methods used and appropriateness of these methods *(e.g., proportion/%s, RR/OR)*		
63. All systematic and random error adjusted? *(e.g., confounding, effect medication etc.)*		
64. **Notes:**		
**8. Limitation and mitigation strategy**		
	Description as stated in report/paper	Location in text*(page# /fig/table)*
65. Strength		
66. Limitation		
67. Strategies to overcome the limitation		
68. Notes:		
**9. Conclusion and other information**		
	Description as stated in report/paper	Location in text *(page#/fig /table)*
69. Key conclusions of study authors		
70. **Notes:**		
**10. Risk of bias (Quality Assessment)**		
External/Internal Validity<br><br>(Note: some criteria would be overlapping with what you have reported in earlier sections. So, please report again to get quick understanding of the quality of the paper)	Often it would not be stated directly in the paper. So, data extractors is/are requested to find information and sate (Yes/No)	Location in text *(page#/fig /table)*

71. Was the study's target population a close representation of the national population in relation to relevant variables?		
72. Was the sampling frame a true or close representation of the target population?		
73. Was some form of random selection used to select the sample, OR was a census undertaken?		
74. Was the likelihood of nonresponse bias minimal?		
75. Were data collected directly from the subjects (as opposed to a proxy)?		
76. Was an acceptable case definition used in the study?		
77. Was the study instrument that measured the parameter of interest shown to have validity and reliability?		
78. Was the same mode of data collection used for all subjects?		
79. Was the length of the shortest prevalence period for the parameter of interest appropriate *(last two weeks or life time prevalence etc. please specify exact period over which symptoms were asked?*		
80. Were the numerator(s) and denominate or(s) for the of interest parameter appropriate?		
81. Notes		

Table S3. Summary of Included studies.

Reference	Study Aim	Study Design	Participants	Data collection	Outcome	Analysis method	Main findings /Conclusion	Quality Ranking
1- Abud and Gaiva [35] Brazil	To analyse the input of growth and development data in the Child Health Handbook.	Cross-sectional study	950 mothers /guardians of children 0-1 year	Questionnaire and direct observation	Rate of growth and development records	Prevalence ratio and chi-square	- 95.4% development data are missing/incomplete - 79.6% of data in growth charts are incomplete/ missing	High
2- Aiga et al. [32] Vietnam	To estimating changes in pregnant women's behaviour towards the frequencies of ANC use and exclusive breastfeeding practices using the MCH Handbook intervention	Cross-sectional study	810 mothers	Structured interview and focus group discussion	Change in attitude toward antenatal care and exclusive breast feeding	SPSS v22, R v3.2.2, Chi-square	- No significant difference in knowledge about need for ≥ three antenatal care visits - increased in proportion of women with ≥ three ANC visits - increased in knowledge & practice of exclusive breast feeding for first six months	High
3- Bhuiyan et al., [21] Bangladesh	To develop the maternal and child health (MCH) handbook and assess its output and utilization among selected pregnant women	Repeated cross-sectional survey	600 pregnant women	Focus Group Discussion FGD, informants meeting, Observations, interview, survey	Improvement rate between pre-intervention variables and post-intervention (MCH book)	SPSS v10, chi-square	- Focus Group Discussion emphasised the need for including MCH handbook in Bangladeshi MCH programs -MCH handbook improve knowledge, practice and utilisation of MCH services	Medium
4- Campbell and Halleran [36] UK	To estimate how often the parent held record PHR is forgotten or lost by parents, to find out what use parents/professionals made of the PHR	Cross-sectional survey	231 Parents 84 health professionals 104 Child health records	Questionnaire survey of health professionals and parents' interview	Rate of retention of the PHR, input of data by parents and professionals and their views	Simple percentages with the help of Epi-info5	-Parent-held record is valued by parents and promote partnership between parents, children and professionals. -Parents read the record (55-87% of pages) and completed input of information (42-74% of pages).	Medium
5- Clendon & Dignam, [31] New Zealand	This paper is a report of a study of the role and impact of the child health and development record book in New Zealand society since its inception	Oral History approach /survey	35 parents (including nurses who are mothers)	Interviews	Narrative experiences of parents toward using a child record book	Narrative synthesis using NVivo software	-Mothers read, reflect upon and reminisce with child health and development record books for generations. -The book plays an important role in the relationship between mother and nurse	Medium
6- Dagvadorj et al. [37] Mongolia	To evaluate handbooks effectiveness on child development in Mongolia. A three-year follow-up of a cluster randomised con- trolled trial (RCT) was conducted.	Cluster RCT	501 mothers	Mongolian Rapid baby scale	Risk of developmental delay	Odds ratio using generalised estimation equation	-Handbook had protective effect on the risk of cognitive delay -Poor cognitive development was increased by higher maternal age, maternal depression one month after	High

							delivery/pregnancy complication	
7- Emond [38] UK	To evaluation the accuracy and completeness of a PHCHR used as part of a prospective study of infant health.	Cross-sectional study	360 parents/PHCHR	Direct observation of records and interview of mothers	Rate of completeness of records in the PHCHR	SPSS-X and chi-squares	-About three-quarters (73.2%) of all consultations were recorded. -Many parents reported a reluctance on behalf of doctors to write in the PHCHR.	Medium
8- Grovdal et al. [22] Norway	To study the effects of a parent-held child health record (PHCHR) that was created by the Norwegian Board of Health with the purpose of introducing this to the whole country	RCT	309 parents	Structured Questionnaire	Change in healthcare utilisation, parent-professional communication and knowledge about child matters	SPSS v12, chi-square	PHCHR was well accepted by parents and professionals but it had no effects on collaboration, healthcare utilization, or other measures that could justify the costs of introducing the record into common use	High
9- Hagiwara et al. [23] Palestine	To examine the effect of Maternal and Child Health (MCH) handbook on women's knowledge and behavior in the Jericho and Ramallah Governorates of Palestine	Repeated Cross-sectional survey	670 women	Questionnaire and focus groups	MCH handbook effect on knowledge and attitude towards MCH care	Difference-in-difference regression analysis	-Knowledge related to MCH increased among MCH handbook users. -The MCH handbook may be an effective tool for communication with health providers and husbands during their first pregnancy	High
10- Hamilton and Wyver [33] Australia	To explore parental use and views of the Child Personal Health Record CPHR	Cross-sectional survey	120 mothers	Questionnaire and interview	Views and attitude toward use of CPHR	Pearson's correlation and ANOVA	-Perceived value of the CPHR was at its highest when the child was younger/first-born. -The CPHR is used by medical professionals, yet broadening its use may increase efficiency of information transfer and promote parent understanding of developmental records	Medium
11- Hampshire et al. [39] UK	To explore variation in use of the PCHR made by mothers, compare heath visitors' and general practitioners' (GPs') use of the PCHR, and to compare health visitors' and GPs' perceptions of the PCHR	Cross-sectional study	534 parents 28GPs 27 Health visitors	Questionnaires and interviews	Variation in use and perceived usefulness of PCHR	Linear regression	-Teenage and first-time mothers use PCHR more than others. -Mothers, health visitors and GPs reported that mothers took the PCHR to baby clinic more frequently than when seeing their GP -Health visitors wrote in the PCHR more frequently than GPs	High

12- Harrison et al. [40] South Africa	To describe the opinions of health personnel and parents at child health clinics in Cape Town; to determine the accuracy and completeness of data recorded on the present 'Road-To-Health' (RTH) card; and to ascertain the views of clinic staff and mothers regarding what information they would like to record.	Cross-sectional study	35 health professionals (nurses) 150 mothers/care givers 150 records (RTH)	Interviews	Views of health personnel and mothers (about child record). Rate of neonatal data, immunisation schedules, measurements, and weight-for-age chart records	Simple percentages	-Most nurses supported the concept of the health card but a large majority recommended a notebook. -A significant proportion of health personnel did not know how to use the weight-for-age chart. -Most mothers attending clinics carried the card, but this number dropped for hospital visits and consultations with private doctors.	Medium
13- Hikita et al. [41] Mongolia	This study investigated the use of a Maternal and Child Health (MCH) handbook, and related factors, in Mongolia	Cross-sectional study	716 women	Interview and questionnaire	Frequency of reading items and recording information in the MCH handbook	SPSS 24 T-test, Chi-square and Odds ratio (multiple regression)	Women's literacy levels, educational attainment, economic status and effective explanation of its usage must be considered in order to enhance the handbook's effectiveness	High
14- Jeffs et al. [42] Australia	To evaluates retention and use of Personal Health Record (PHR) among parents and professionals	Cross-sectional study	622 parents 458 PHR 911 professionals	Questionnaires	Rate of retention of PHR by parents and frequency of use by parents and professionals	SAS package and EPI INFO v5	-78% retention of record at four years. (inspection) - 91 % PHR had at least one immunization recorded while 68% had a complete regimen. -Overall, 93% of parents expressed satisfaction with the PHR, while 64% of all health care providers also felt that the PHR was beneficial.	High
15- Jessop et al. [43] Ireland	To examine prospectively three sets of immunisation information data (Parent Held Child Health Record-PHCHR, Health Service Executive-HSE & parent recall) on the same children to assess the relative standards of information, particularly parental recall	Linkage study	1070 mothers 307 PHCR	Questionnaires	Matching of immunisation records from three sources	kappa statistics were calculated using STATA 9.2.	-The agreement of primary immunisations between PHCHR and HSE or parental recall is 74-78% and for HSE and parental recall is 93% but kappa scores are not statistically significant. -93% agreement between the parents records and HSE records for MMR (kappa = 0.42, p<0.001)	High
16- Kawakatsu et al. [44]	To clarify the effectiveness of and identify the factors related to possession of an MCH handbook among parents in	Cross-sectional survey (with study & control groups)	1983 mothers	Questionnaire	Rate of child immunisation, effect on mothers' knowledge &	Epi Info v3.5, PSM, multivariate	Impacts of 5.9, 9.4, and 12.6 percentage points for higher health knowledge and for proper health-seeking behavior	High

Kenya	rural Western Kenya using propensity score matching (PSM)				attitude towards child fever/diarrhea	analysis and Odds ratio	for fever and diarrhea, respectively, were statistically significant.	
17- Koh et al. [45] Singapore	To investigate parents' perceptions of developmental checklists and the child development monitoring schedule in the Singapore health booklet	Cross-sectional study	450 parents	Interview	Perception of parents from reading/filling the booklet as well as rate of completeness of each domain of the book	Mann–Whitney U tests or x^2 tests	-About half of parents attempted the checklists with minimal help from professionals. -7 in 10 parents of children with developmental concerns found the checklists useful -1 in 4 parents brought their child for a two to three years developmental monitoring visit	Medium
18- Lakhani et al. [46] UK	To evaluate efficiency and effectiveness of home-based health booklet	RCT	450 mothers 140 booklets	Interviews and Questionnaires	Views of parents & professionals about the booklet, uptake of immunisation /developmental assessment and number of entries	Descriptive statistic using percentages, mean and median	-Evaluation was unable to show any effect of the record on immunisation and developmental assessment uptake - Its value in improving communication between the numerous health and other care agencies was dependent on its proper use	Medium
19- Miller [24] Germany	To evaluate a trial of parent-held child health record in the armed forces?	Cohort	137 families 123 records	Interview	Families views about the scheme and information input in the record	Simple percentages and proportion	-90% records are kept for up to one year -45% favour its introduction on wide scale -72% developmental and 74% immunisation records are made in the booklet	Medium
20- Mukanga and Kiguli [47] Uganda	To explore and identify factors affecting child health card use and retention, and its effects on immunization	Cross-sectional study	260 household	Interview	factors affecting child health card retention, the effect of card retention on immunization, as well as its use by mothers and caretakers	Epi Info v3.3.3 Odds ratio	-66% children had a child health card. -Children delivered at a health facility, whose mothers had a health problem during pregnancy, taken to health facility in last three months were more likely to have a card (AOR 3.74, 2.4, 2.37 respectively). -Children who had cards were 10 times more likely to be fully immunised (OR = 9.55, 95% CI 3.19, 29.45).	High

21- O'Flaherty et al. [25]<br><br>Australia	To evaluate use and views of mothers and professionals about the PHR	Cohort	167 mothers<br>135 records<br>90 professionals	Interview and questionnaire	Parent and professional views and use of the record	Simple percentages and proportions	-92% parent like book and use it frequently<br>-93% community health staff, mainly nurses like and use the book<br>Most private doctors did not find it useful	Medium
22- Osaki et al. [34]<br><br>Indonesia	To examined the effect of MCHH use in rural Java, where service coverage was comparatively low	RCT	454 mothers	Questionnaire	Maternal and child care during pregnancy and child birth	SPSS v18, Generalised mixed model, chi-square, t-test	-intervention effect is significant toward maternal utilisation of MCH services<br>-MCHH influence giving supplements to child (OR=2.03), home care and facilitates child growth	High
23- Palombo et al. [48]<br><br>Brazil	To evaluate the use and records of the Child Health Handbook (CHH), especially growth and development	Cross-sectional	358 mothers<br>185 CHH	Questionnaire	Rate of use and record input in the CHH	Epi Info v6.04, chi square & fisher's exact-test in STATA	-53% mothers take CHH at interview<br>-52% mothers said were instructed to always take the CHH<br>-annotations in CHH were made in 49%	Medium
24- Polnay and Roberts [26]<br><br>UK	To evaluate the booklet before it is introduced to all population	Cohort study	67 mothers	Interview	Use of booklet and record input by parents	Simple percentages and proportions	-97% vaccination records completed<br>-Book is well used by majority of parents<br>-80% having read all book at months<br>-70% retain the book at 1year	Medium
25- Price et al. [27]<br><br>UK	To evaluate different aspects of the Parent held record (PHR), mainly client and professional views of its success and appropriateness	Cross-sectional study	253 parents<br>51 health professionals	Interview and questionnaire	Parents and professionals' views of success and appropriateness of PHR	Simple percentages and proportions	-21.6% had written in the PHR<br>-53.4% found it easier to ask questions because they hold the PHR<br>-86.9% parents said health visitors are most likely to fill record<br>-21.6% GPs wrote in the book	Medium
26- Saffin and Macfarlane [28]<br><br>UK	To evaluate parent use, loss/forgotten rate and completeness of parent held record PHR	Cross-sectional study	252 parents	Questionnaire	Parents use/views of PHR and completeness of record input	Simple percentages and proportions	-parents like the scheme of PHR and usually remember to take it to clinics<br>- PHR were more likely to be completed than clinic based records	Medium

27- Stacy et al. [29]<br><br>USA	To evaluate satisfaction, use, behaviour change and barriers to use of child record (Passport)	Cross-sectional study	100 mothers	Questionnaire	Views of parents with respect to use of the passport	Mean and percentages	-Patients reported high levels of satisfaction with all applicable use of the record<br>-Respondents believed the record was a useful tool that served as a cue to increase their action in health seeking behaviours.	Medium
28- Tarwa and De Villiers [49]<br><br>South Africa	To assess whether the RTHCs are completed and interpreted adequately at the primary, secondary and tertiary care levels in South Africa.	Cross-sectional study	300 parents	Questionnaire	Use of RTHC	Percentages and chi-square using Epi INFO 6	-48% of the consultations; of these respondents<br>-about 72% thought that bringing along the RTHC was not necessary<br>-majority of health workers do not ask about the RTHC	Medium
29- Troude et al. [50]<br><br>France	to assess, in individual children, the validity of Apgar scores reported in the PCHR using maternity medical records as the gold standard.	Linkage study	392 mothers	Questionnaire	Sensitivity and specificity of PCHR-reported Apgar scores compared to maternity record	Percentage and Chi-squares	- proportion of missing Apgar scores in the PCHR was higher when scores in the medical records were lower<br>- Apgar scores reported in the PCHR were overestimated when scores in the medical records were low	High
30- Walton and Bedford [51]<br><br>UK	To explore parental views of the 'new' PCHR, their experiences in receiving it, and its subsequent use, focusing on specific issues of current debate among health professionals	Cross-sectional study	89 parents	Questionnaire	Parents views and use of the new PCHR	Simple percentages and proportions	-98% reported they used the PCHR<br>-92% reported they 'always' took it with them when seeing healthcare staff<br>-health visitors were more likely to use and refer to the record	Medium
31- Wright and Reynolds [52]<br><br>UK	To evaluate use of re-designed personal child health record (PCHR) compared to old record	Cross-sectional	1369 mothers	Questionnaire	Parents views and use of the PCHR	Percentages and chi-square test	-over 90% parents found both records easy to read<br>-health visitors (about 90%) use both records more than other professional<br>-over95% of both records were used in Baby clinics	High
32- Yanagisawa et al. [30]<br><br>Cambodia	To evaluate the impact of the MCH handbook on maternal knowledge and behaviour	Quasi - experimental	640 mothers<br>18 health staff	interview	Rate of antenatal care (ANC) attendance, delivery with skilled birth attendants (SBAs) and	Percentage, Difference in Difference (DID), OR	- DID analyses revealed that all key indicators increased in the intervention group<br>-MCHH increased maternal ANC attendance, delivery with	High

		delivery at a health facility	SBAs and delivery at a health facility.

Note: PHCHR: Parent-held child health record, Health Service Executive-HSE.

Table S4. Parent use and views of PHCHR.

Outcomes	Reference	Records	Effect size/comments
Record book possession/ retention	[21]	MCHH	99.5% retention rate at one year
	[36]	CPHR	77% retention rate at six months
	[38]	CPHR	96% retention rate (period unknown)
	[42]	CPHR	93% claim retention after five years, and 78% present it (rate decrease with increase in child age, $p < 0.00001$)
	[46]	CPHR	93% of study group retained at one year
	[24]	CPHR	89.7% retention rate at six months & 62% at one year
	[47]	CPHR	66% retention at two years (influenced by delivery in health facility AOR3.74, previous pregnancy complications AOR2.4, child who previously visited a facility AOR2.37, $p < 0.05$)
	[34]	MCHH	70.5% ($p < 0.001$) intervention 9.6% control retain and present to interviewers after two years (R = 69.7)
	[26]	CPHR	90% parents retain record at three months & 70% at 12 months
	[49]	CPHR	70% of those at a primary care facility, 32% at secondary facility, 42% at tertiary facility did not have their records at the time of visit. This difference was highly significant ($p = 0.0000002$). (period unclear)
Satisfaction with prior explanation received	[36]	CPHR	90% considered explanation adequate
	[33]	CPHR	57.5% think is adequate, 15% require more, 20.8% no explanation received
	[34]	MCHH	74.3%, p<0.001 (intervention), 18.1%, p<0.001 (control) mothers receive explanation from health personnel (R=60.1%)
	[51]	CPHR	53% received adequate explanation and another 25% were familiar with it, while 16% will like more
Read content/ information from book	[36]	CPHR	84% read other information from the book and found useful
	[23]	MCHH	Reading habit increase from none (t-test=2.96, $p \leq 0.01$), to primary (t-test =5.17, $p \leq 0.01$) and secondary education (t-test=7.65, $p \leq 0.01$).
	[21]	MCHH	84.2% mothers read all content (not significant with age, family income & educational level)
	[41]	MCHH	88.1% read handbook often/very often - influenced by educational level (AOR = 2.52 middle, 3.19 high), wealth index (AOR 2.82 middle, 6.29 high) and prior explanation received (AOR=2.42), $p < 0.05$
	[42]	CPHR	85% read parenting & first aid information
	[45]	CPHR	90.7% read the developmental checklists section, mostly before schedule visits (49.8%)
	[34]	MCHH	65% $p < 0.001$ (Intervention) 15.9% $p < 0.05$ (control) mothers read the MCHH (R = 54.2)
	[26]	CPHR	80% read all book, 15% read some of it, and 5% looked at the pictures at three months
	[29]	CPHR	Read to get important information (X = 3.63), find important phone numbers (X = 3.54)
Take record book to child clinic/ regular child check-ups	[36]	CPHR	94% always/usually
	[22]	CPHR	60% always/usually
	[33]	CPHR	92.5% mother always/usually (associated with taking the PCHR to GPs, r = 0.239, $p = 0.009$, but not influenced by media $p = 0.099$ or child's age $p = 0.084$)
	[39]	CPHR	96.4% mothers always/usually (17.3% more, 95% CI, $P < 0.0001$ compared to GPs visit)
	[40]	CPHR	100% in public hospitals & 84% in private
	[42]	CPHR	85% always/usually
	[46]	CPHR	85% always
	[27]	CPHR	*15%, 55%and 75% parents respectively attend the clinic with the CPHR over three data collection periods
	[28]	CPHR	94% always/usually
	[52]	CPHR	Parent using both old and new versions of book took it more than twice to clinic (95.7% old, 95.6%, 96.4% New)
Take record book to other child consultations e.g., General Practitioner (GP), Private doctor, specialists visits	[36]	CPHR	81% child surveillance clinic and 29% GP surgery
	[22]	CPHR	19% general practice, 15% specialist/hospital or 12% other health personnel
	[33]	CPHR	63.3% mothers always/usually (influenced by media, r=0.193, $p = 0.035$, number r = 0.261 $p = 0.004$ & age of child r = -0.296 $p = 0.001$)
	[39]	CPHR	79.2% mothers always/usually
	[40]	CPHR	57% hospital visits, 34% private doctor, 62% private hospital
	[42]	CPHR	45% always/usually
	[29]	CPHR	Take record to clinics other than their primary provider (X=3.43)
	[52]	CPHR	More parents with old record took to family doctor than with new record (63.3% old, 52.2%, 53.5% New, $p = 0.007$)
	[21]	MCHH	83.3% always take records (not significant with age, family income & educational level, $p > 0.05$)

Take record to general health facility/ hospital visits (baby clinic/ GPs/Specialists)	[34]	MCHH	54.6% $p < 0.001$ (intervention) vs 11.8% $p < 0.05$ (control) mothers brought MCHH to more than two facilities (R = 45%)
	[48]	CPHR	79.6% mothers take handbook to health appointments. 53.2% take it at the time of the interview, influenced by (p<0.05) mother being instructed to take it (61.9%), mother observed health professionals making annotations (68.7%), mother being informed of the weight gain/height of the child (88%) and child age of ≤1year (66%), but not significant with child nutritional status/adequate development ($p > 0.05$)
	[29]	CPHR	Take record to the healthcare provider at each visit (X = 4.02).
	[51]	CPHR	92% always & 8% on most occasions took the red book with them when seeing healthcare staff, particularly for contact with health visitors (98%), immunization (95%), family doctor/GP (93%), hospital (89%), casualty department (78% and dentist (53%)
Frequency of putting information by parents	[33]	CPHR	36.7% never, 40.8% usually, 7.5% only when suggested by a professional
	[39]	CPHR	80.2% mothers always/usually
	[21]	MCHH	76.1% record all (not significant with age, family income & educational level, p > 0.05)
	[41]	MCHH	59.8% often/very often record information – influenced by educational level (AOR = 1.90 middle, 2.16 high), prior explanation received (AOR = 2.57), but less likely for those with congenital/chronic diseases $p < 0.05$. not significant with wealth index
	[45]	CPHR	33.8% complete the developmental checklists without assistant, 11.3% complete, but verify from doctor/nurses, 29.8% had nurses/doctors complete it by interviewing them, 18,7% had nurses/doctors test the child and complete it while 6.4% can't remember
	[46]	CPHR	70% made entries themselves
	[25]	CPHR	28% parents had written in the progress note
	[27]	CPHR	21.6% had written at least three times, while 17% never written anything
	[29]	CPHR	Always write information as advised (X = 4.19)
	[51]	CPHR	43% of mothers or their partner had written in it (37% English speakers compared to 69% non-English speakers, $p = 0.04$)
	[52]	CPHR	Similar record input in old and new record versions (55.5% old, 65.9%, 47.6% New)
Perceived general usefulness	[36]	CPHR	74% parents think it's very important & 89% like it
	[31]	CPHR	Qualitative evidence from 35 parents consider record book important for childcare and building relationship with nurses
	[22]	CPHR	41% said it increase awareness of child health and development and 38% said it makes it easier to talk to their health providers about the child
	[39]	CPHR	82.5% felt is good & 92.1% felt useful (significant with teenage mother p0.001 & first-time mother p0.002), particularly in understanding child health/development (63.2%)
	[21]	MCHH	78% mothers perceived positive usefulness against 22% negative
	[40]	CPHR	64% considered record as very important & 85% perceived infant growth adequate
	[42]	CPHR	27% feeling of usefulness in 0-12months group to 5% among 49-50 months age group (decrease with age, $p < 0.001$)
	[45]	CPHR	92.7% indicated the developmental checklists as useful for checking child's development & 66.7% in identifying children with developmental concerns (more understanding of checklist among parents of children with 'No concerns' 91.0% than with concerns-79.7%, U= 15663, $p = 0.003$)
	[46]	CPHR	97% mothers like it
	[24]	CPHR	69% like it
	[25]	CPHR	92% like the record & 87% would like it for future children
	[26]	CPHR	65% value the book, against 10% who said the book is of no value
	[27]	CPHR	53.4% found it easier to ask questions because they hold the PCHR
	[28]	CPHR	75% of parents who had experience the CPHR thought they should hold it compared to 26% of parents who didn't
	[29]	CPHR	Patients reported they were very likely to stay informed about their children's health (X =4.30), visit the doctor as recommended during pregnancy (X = 4.29), keep baby shots up to date (X = 4.28), remember their healthcare appointments (X = 4.16) and stay informed about her own health (X = 4.08). Patients were somewhat less likely to call the doctor when they thought they themselves were sick (X = 3.97)
	[49]	CPHR	Major reason for not bringing the child record was perceived non-importance for the visit (53/70 primary, 20/32 secondary, 33/42 tertiary health facilities)
	[51]	CPHR	All (100%) strongly agree/agree the record is helpful. 71% said it help their understanding of the child, majority thought the illustrations (98%) and information (96%) were right.

Satisfaction with record	[22]	CPHR	65% were satisfied with having the record and 92% were in favour of introducing it permanently, particularly parents of children with developmental concern
	[29]	CPHR	Patients reported being very satisfied with record's sturdiness (X = 4.05) and ease of reading (X = 4.03); while they reported slightly less satisfaction with the organization (X = 3.99), ease of locating information (X = 3.85), and ease of understanding all of the Passport words (X = 3.81).
	[52]	CPHR	Parents using old and new version of record rated it as easy to read (97.1% old, 95.7%, 98.2% New), bright and colourful (67.9% old, 63.7%, 78.7% New, p0.002), attractive (64.6% old, 60.2%, 77% New, $p < 0.001$)
	[30]	MCHH	All mothers interviewed (20) were satisfied with handbook due to its appearance, practical information, convenience, long-term value, size and illustrations.
Most valued/ frequently used section	[33]	CPHR	40% growth chart (influenced by number of children r = -0.261 $p = 0.004$) compared to 23.3% immunisation record or 21.7% child health/development notes (all influenced by interacting with people about infants; and a smaller number of children for growth/development, $p < 0.05$)
	[39]	CPHR	36.8% weight/growth chart compared to 31.7% development record, 18% advice/information, 16% all, 14.7% immunisation
	[40]	CPHR	88% weight & immunisation sections
	[42]	CPHR	36% immunization, 29% development, 16% progress notes
	[28]	CPHR	Parents like to watch their child's weight (35%), use information for reference (15%) and see what is written generally (11%)
	[51]	CPHR	98% reported using the PCHR as a record of their child's health and development. Fewer used it as a source of information (41%), remainder for appointment date (28), record of contact with professionals (45%) or a keepsake (33%)
Increase Male participation	[23]	MCHH	Finding from FGD with 32 women reported increase in male participation during pregnancy and child care
Overcome rumours	[23]	MCHH	Some women (unspecified) from FGD with 32 women reported the record book in helping them deal with pregnancy rumours
Major Concerns and/or recommendations	[21]	MCHH	59.3% mothers suggested more illustrations with colour pages
	[40]	CPHR	52% did not comprehend weight chart, 47% immunisation and 73% milestone section
	[22]	CPHR	89% parents said PHCHR would be used more if professionals show interest
	[45]	CPHR	Less parents of children with "No concerns" subsample (55.9%) indicated that pictures could help them understand the developmental checklists better, compared to parents "With Concerns" (71.0%) U = 15,449, $p = 0.015$)
	[29]	CPHR	24% reported not being asked by clinic to present record
	[52]	CPHR	Too few illustrations (29.9% old record, 25.1%, 31.4% New records, $p = 0.1$)
General record input by health facilities/ professionals (unspecified)	[33]	CPHR	15% mothers said always
	[40]	CPHR	New information always entered in public clinics (100%) than private (64%)
	[34]	MCHH	42.1% (intervention) vs 8.9% MCHH (control) filled in by more than two personnel (R = 33.7%)
	[48]	CPHR	49% mothers observe professionals making entries
	[49]	CPHR	13%, 16%, 50% were respectively asked during previous consultation visits against 56%, 32% and 50% who were not asked, in the primary, secondary and tertiary care settings respectively
Health visitors frequency of record input and use	[39]	CPHR	94.4% mothers said always/usually (37.6% more, 95% CI, $P < 0.0001$ compare to GPs).
	[27]	CPHR	86.9% parents said health visitors are most likely to fill information in the record compared to hospital doctors
	[51]	CPHR	100% health visitors
	[52]	CPHR	Significant record input in both old and new version of record (87.7% old, 91%, 96.4% New)
GPs/doctors frequency of record input and use	[33]	CPHR	87.5% and 5.8% mothers respectively indicated GPs and casualty doctors' likelihood to use record
	[39]	CPHR	56.9% mothers said always/usually
	[40]	CPHR	14% ever asked by private doctors
	[27]	CPHR	12.9% likely to use record
	[51]	CPHR	67% GPs/family doctors, 31% paediatrician and 8% casualty doctor
	[52]	CPHR	Slightly more input in old record than new (27.2% old, 17.1%, 19.4% New)
Nurses/Midwives frequency of record input and use	[33]	CPHR	92.5% mothers said baby nurses more likely to use
	[42]	CPHR	80% always make entry during child check-ups

	[51]	CPHR	66% midwife, 47% children nurse
	[52]	CPHR	Similar record input in both old and new records by midwives (50.7% old, 53.3%, 50% New)
Others use/input	[33]	CPHR	35% mothers indicated other professionals likely to use record
Professionals referral to previous record	[40]	CPHR	84% public clinic staff, than private clinics (46%)
	[51]	CPHR	95% health visitors were more likely to refer to the record compared to 69% family doctor/GPs, 54% children's nurse, 53% paediatrician and 23% casualty department doctor

CPHR=Child Personal Health Record, MCHH=Maternal & Child Health Hand, R = Point difference using 'Difference-in-Difference' analysis, OR= Odds Ratio, AOR=Adjusted Odds Ratio, U= Mann–Whitney test, r= correlation coefficient, X= mean, with a range from lowest-1 to highest-5. *= Figure estimated from graph

Table S5. Record input measured by direct observation.

Record Outcomes	Reference	Records	Rate of completeness/comments
Birth details/ baseline	[36]	CPHR	56% fully completed birth details, 42% partial & 2% none. 44% fully completed family history
	[40]	CPHR	100% (date of birth, birth weight, discharge data), 98% (head circumference, APGAR), 83-100%% maternal/pregnancy data
	[42]	CPHR	90% birth weight, 86% APGAR
	[46]	CPHR	100% surname, 93% address, 10% photographs, 85% time of birth, 96% birthweight, 79% gestation, 84% delivery, 2% allergies, Important names/phone number (9-62%)
	[48]	CPHR	48% complete Child identification, 47.5% complete Information about birth
	[26]	CPHR	100% birth weight
	[28]	CPHR	8% CPHR and 18% Clinic Held Record had birth information left blank
	[50]	CPHR	1-min Apgar score was missing in 7.9% ($n = 26$) of CPHR and the 5-min score in 6.0% ($n = 20$). When the 1-min Apgar score noted in medical records was ≥7, 5.0% ($n = 15$) of 1-min Apgar scores were missing in the CPHR versus 37.9% ($n = 11$) when the Apgar score in the medical records was <7 ($p < 0.001$). Similarly, 5-min Apgar scores were missing in 5.5% ($n = 18$) of CPHR when the score noted in the medical records was ≥7 and in 66.7% ($n = 2$) of CPHR when the score was <7 in the medical records ($p = 0.01$).
Growth/ weight data /chart	[35]	CPHR	79.6% incomplete record (incompleteness associated with increased child age, PR = 1.34, $p = 0.00$)
	[36]	CPHR	39% parents' section and 32% professionals section completed
	[40]	CPHR	45% public & 66% private clinics completed charts
	[42]	CPHR	58% plotted on percentile charts
	[46]	CPHR	74% with at least one entry (mean 5.8), overall accurateness of weight chart is 52%
	[47]	CPHR	75% weight plotted
	[48]	CPHR	33.8% Anthropometric measurements record sheet, 8.9% completed Height chart, 8.9% completed Weight chart
	[28]	CPHR	About 75% CPHR and 50% clinic held record had up to 90% weight chart plot
	[49]	CPHR	Weight was plotted more at tertiary (36%) and secondary health facilities (27%) than primary (14%) (chi square = 12.82; $p = 0.002$)
Developmen t data/chart	[35]	CPHR	95.4% incomplete record (incompleteness associated with number of children PR = 1.03, $p = 0.034$)
	[36]	CPHR	Only 19% completed
	[40]	CPHR	12% public & 8% private clinics completed milestones input
	[42]	CPHR	50% completed by parents, 11% partially
	[45]	CPHR	Rate of developmental checklist completed at 4-8weeks (42.9% complete, 48.4% incomplete), 3-5 months (51.6% complete, 74% incomplete), 6-12months (38% complete, 62.2% incomplete), 15-18months (43.6% complete, 69.3% incomplete), 2-3years (14.9% complete, 24.4% incomplete). Attendance at scheduled child development monitoring visits are 61.3% (4-8weeks visit), 87.8% (3-5months), 78.9% (6-12months), 78.4% (15-18months) and 28.7% (2-3years)
	[46]	CPHR	Milestones recorded include smiled (50%), sat (32%), walked (2%), one word (4%), teeth (13%) and others (11%) (significant with English as first language $p < 0.025$), 46% general first assessment, 16% second assessment
	[24]	CPHR	72% completed
	[48]	CPHR	8.3% completed Development chart
	[26]	CPHR	59% first smile, 59% lifted head, 37% crawling, 25% walking
	[28]	CPHR	93% CPHR and 91% Clinic Held Record (CHR) had 2months development checks. 8% CPHR and 27% CHR had no eight months development checks recorded. 69% checklist were fully/partially completed
Immunisatio n /Vaccination schedules	[36]	CPHR	40% fully completed
	[40]	CPHR	100% recorded
	[42]	CPHR	68% had all records, while 91% had at least 1
	[43]	CPHR	64.8% complete record (93% agreement with national record-HSE for measles, mumps and rubella-MMR (kappa = 0.42, $p < 0.001$). Compared to HSE, CPHR underestimated immunisation by 17.4% while parent recall at five years overestimated by 2%.

	[46]	CPHR	20% first immunization, 17% second immunization, 12% third immunization recorded
	[24]	CPHR	74% recorded
	[25]	CPHR	75% cases had accurate record of immunisation
	[48]	CPHR	96.6% completed Vaccination scheme
	[28]	CPHR	96% in CPHR and 93% clinic record
	[49]	CPHR	80% coverage at primary and 82% at secondary facilities
Child clinic findings/ assessment results	[36]	CPHR	44% fully completed (general)
	[40]	CPHR	Public-private clinic input for baby serology (3%-6%), Hearing test (3%-8%), Vision test (2%-6%)
	[42]	CPHR	57% hearing questions completed
	[25]	CPHR	91% records had progress notes made by clinic nurses, 18.5% had notes made by doctor
	[28]	CPHR	61% hearing and 55% vision checklists were fully or partially completed
Child consultations	[36]	CPHR	5% completed, 13 partially & 82% not completed by doctors. 66% 6-8weeks check and 36% three months check fully completed
	[42]	CPHR	Out of 56% children taken to doctor, only 18% recorded. 2% out of 9% emergency contacts recorded
	[28]	CPHR	77% CPHR compared to 60% Clinic Held Record had prescriptions recorded
Antenatal care	[32]	MCHH	76.2% take book for ANC, only 66.3% had at least three ANC visits recorded by health staff
Family planning	[40]	CPHR	69% public clinic, 0% private
Breast Feeding	[32]	MCHH	76% present records, only 68.1% had several boxes checked for EBF (unspecified) by mothers
	[42]	CPHR	49% parents recorded
General record input	[21]	MCHH	76.1% of mothers record all required information
	[38]	CPHR	73.2% of all records completed by professionals/parents (incompleteness associated with African/Caribbean origin and poor families, $p < 0.05$ but not with pre-term birth)
	[25]	CPHR	75% have all/most of the records completed (≥9 entries),
	[28]	CPHR	9 items were more completed in the CPHR, two items more in clinic record and four items no differences

CPHR=Child Personal Health Record, MCHH=Maternal and Child Health Hand, PR=Prevalence Risk.

References

1. Volkmer, R.E.; Gouldstone, M.A.; Ninnes, C.P. Parental Perception of the Use and Usefulness of a Parent-held Child Health Record. *J. Paediatr. Child Health* **1993**, *29*, 150–153, doi:10.1111/j.1440-1754.1993.tb00470.x.
2. Takeuchi, J.; Sakagami, Y.; Perez, R.C. The Mother and Child Health Handbook in Japan as a Health Promotion Tool: An Overview of Its History, Contents, Use, Benefits, and Global Influence. *Glob. Pediatr. Heal.* **2016**, *3*, doi:10.1177/2333794X16649884.
3. Mudany, M.A.; Sirengo, M.; Rutherford, G.W.; Mwangi, M.; Nganga, L.W.; Gichangi, A. Enhancing Maternal and Child Health Using a Combined Mother & Child Health Booklet in Kenya. *J. Trop. Pediatr.* **2015**, *61*, 442–447, doi:10.1093/tropej/fmv055.
4. Kusumayati, A.; Nakamura, Y. Increased Utilization of Maternal Health Services by Mothers Using the Maternal and Child Health Handbook in Indonesia. *J. Int. Heal.* **2007**, *22*, 143–151, doi:10.11197/jaih.22.143.
5. Mori, R.; Yonemoto, N.; Noma, H.; Ochirbat, T.; Barber, E.; Soyolgerel, G.; Nakamura, Y.; Lkhagvasuren, O. The Maternal and Child Health (MCH) Handbook in Mongolia:A Cluster-Randomized, Controlled Trial. *PLoS ONE* **2015**, *10*, e0119772, doi:10.1371/journal.pone.0119772.
6. McMaster, P.; McMaster, H.J.; Southall, D.P. Personal Child Health Record and Advice Booklet Programme in Tuzla, Bosnia Herzegovina. *J. R. Soc. Med.* **1996**, *89*, 202–204.
7. Shah, P.M.; Selwyn, B.J.; Shah, K.; Kumar, V.; Abraham, S.; Akhter, M.S.; Alahakone, K.; Bacalzo, F.T.; Fall, M.; Hammamy, M.T.; et al. Evaluation of the Home-Based Maternal Record: A WHO Collaborative Study. *Bull. World Health Organ.* **1993**, *71*, 535–548.
8. McElligott, J.T.; Darden, P.M. Are Patient-Held Vaccination Records Associated With Improved Vaccination Coverage Rates? *Pediatrics* **2010**, *125*, e467–e472, doi:10.1542/peds.2009-0835.
9. Fujimoto, S.; Nakamura, Y.; Ikeda, M.; Takeda, Y.; Higurashi, M. Utilization of Maternal and Child Health Handbook in Japan. *Nihon Koshu Eisei Zasshi* **2001**, *48*, 486–494.
10. Garg, P.; Ha, M.T.; Eastwood, J.; Harvey, S.; Woolfenden, S.; Murphy, E.; Dissanayake, C.; Jalaludin, B.; Williams, K.; McKenzie, A.; et al. Explaining Culturally and Linguistically Diverse (CALD) Parents' Access of Healthcare

Services for Developmental Surveillance and Anticipatory Guidance: Qualitative Findings from the 'Watch Me Grow' Study. *BMC Health Serv. Res.* **2017**, *17*, 228, doi:10.1186/s12913-017-2143-1.

11. Hooker, L.; Williams, J. Parent-Held Shared Care Records: Bridging the Communication Gaps. *Br. J. Nurs.* **1996**, *5*, 738–741, doi:10.12968/bjon.1996.5.12.738.
12. Riverin, B.; Li, P.; Rourke, L.; Leduc, D.; Rourke, J. Rourke Baby Record 2014: Evidence-Based Tool for the Health of Infants and Children from Birth to Age 5. *Can. Fam. Physician Méd. Fam. Can.* **2015**, *61*, 949–955.
13. Farida, N. Determinants of the mch handbook utilization by pregnant women at wanakerta health center of karawang regency in 2015 Dengan Ibu Hamil Di Puskes. *Southeast Asian J. Midwifery* **2016**, *2*, 33–41.
14. Cohen, S.J.; Gitterman, B.A.; Baron, A.L.; Reiner, K.L.; Lynch, K.R. Improving Adherence with Preventive Pediatric Care Guidelines through the Use of a Parent-Held Child Health Record | Cochrane Library. Available online: https://www.cochranelibrary.com/central/doi/10.1002/central/CN-00598143/full (accessed on 25 November 2018).
15. Calvin, S.D.R. *Maternal and Child Health Handbook among the Indigenous Peoples in the Philippines : The Case of Tagbanua Mothers and Children of Coron Island, Palawan*; Osaka University: Osaka, Japan, 2010.
16. Turner, K.E.; Fuller, S. Patient-Held Maternal and/or Child Health Records: Meeting the Information Needs of Patients and Healthcare Providers? *Online J. Public Health Inform.* **2011**, *3*, 1–48, doi:10.5210/ojphi.v3i2.3631.
17. Baequni; Nakamura, Y. Is Maternal and Child Health Handbook Effective?: Meta-Analysis of the Effects of MCH Handbook. *J. Int. Health* **2012**, *27*, 121–127.
18. PRISMA. PRISMA 2009 Checklist. 2009. Available online: https://journals.plos.org/plosmedicine/article/file?type=supplementary&id=info:doi/10.1371/journal.pmed.1002203.s001 (accessed on 18 July 2018).
19. Centre for Reviews & Dissemination CRD. *CRD Guidance for Undertaking Reviews in Health Care*, 3rd ed.; York Publishing Services Ltd.: Layerthorpe, UK. 2009.
20. World Bank. Countries and Economies 2008. Available online: https://data.worldbank.org/country (accessed on 27 November 2018).
21. Bhuiyan, S.U.; Nakamura, Y.; Qureshi, N.A. Study on the Development and Assessment of Maternal and Child Health (MCH) Handbook in Bangladesh. *J. Public Health Dev.* **2006**, *4*, 45–59.
22. Grøvdal, L.B.; Grimsmo, A.; Nilsen, T.I.L. Parent-Held Child Health Records Do Not Improve Care: A Randomized Controlled Trial in Norway. *Scand. J. Prim. Health Care* **2006**, *24*, 186–190, doi:10.1080/02813430600819769.
23. Hagiwara, A.; Ueyama, M.; Ramlawi, A.; Sawada, Y. Is the Maternal and Child Health (MCH) Handbook Effective in Improving Health-Related Behavior Evidence from Palestine. *J. Public Health Policy* **2013**, *34*, 31–45, doi:10.1057/jphp.2012.56.
24. Miller, S.A. A Trial of Parent Held Child Health Records in the Armed Forces. *BMJ Br. Med. J.* **1990**, *300*, 1046.
25. O'Flaherty, S.; Jandera, E.; Llewellyn, J.; Wall, M. Personal Health Records: An Evaluation. *Arch. Dis. Child.* **1987**, *62*, 1152–1155.
26. Polnay, L.; Roberts, H. Evaluation of an Easy to Read Parent-Held Information and Record Booklet of Child Health. *Child. Soc.* **1989**, *3*, 255–260, doi:10.1111/j.1099-0860.1989.tb00350.x.
27. Price, S.; Fulop, N.; Mills, A. Sharing Information: An Evaluation of a Parent-Held Child Health Record Scheme. *Crit. Public Health* **1991**, *2*, 32–37, doi:10.1080/09581599108406820.
28. Saffin, K.; Macfarlane, A. How Well Are Parent Held Records Kept and Completed? *Br. J. Gen. Pract.* **1991**, *41*, 249–251.
29. Stacy, R.D.; Sharma, M.; William, A.T. Evaluation of the Use of a Parent-Held Child Health Record by Pregnant Women and Mothers of Young Children. *Calif. J. Health Promot.* **2008**, *6*, 138–142.
30. Yanagisawa, S.; Soyano, A.; Igarashi, H.; Ura, M.; Nakamura, Y. Effect of a Maternal and Child Health Handbook on Maternal Knowledge and Behaviour: A Community-Based Controlled Trial in Rural Cambodia. *Health Policy Plan.* **2015**, *30*, 1184–1192, doi:10.1093/heapol/czu133.
31. Clendon, J.; Dignam, D. Child Health and Development Record Book: Tool for Relationship Building between Nurse and Mother. *J. Adv. Nurs.* **2010**, *66*, 968–977, doi:10.1111/j.1365-2648.2010.05285.x.
32. Aiga, H.; Nguyen, V.D.; Nguyen, C.D.; Nguyen, T.T.T.; Nguyen, L.T.P. Knowledge, Attitude and Practices: Assessing Maternal and Child Health Care Handbook Intervention in Vietnam. *BMC Public Health* **2016**, *16*, 129, doi:10.1186/s12889-016-2788-4.
33. Hamilton, L.; Wyver, S. Parental Use and Views of the Child Personal Health Record. *Aust. Educ. Dev. Psychol.* **2012**, *29*, 66–77, doi:10.1017/edp.2012.2.
34. Osaki, K.; Hattori, T.; Toda, A.; Mulati, E.; Hermawan, L.; Pritasari, K.; Bardosono, S.; Kosen, S. Maternal and Child Health Handbook Use for Maternal and Child Care: A Cluster Randomized Controlled Study in Rural Java, Indonesia. *J. Public Health* **2018**, 1–13, doi:10.1093/pubmed/fdx175.
35. Abud, S.M.; Gaíva, M.A.M. Records of Growth and Development Data in the Child Health Handbook. *Rev. Gaúcha Enferm.* **2015**, *36*, 97–105, doi:10.1590/1983-1447.2015.02.48427.

36. Campbell, H.; Halleran, J. An Evaluation of the Personal Child Health Record in Fife. *Health Bull.* **1993**, *51*, 399–406.
37. Dagvadorj, A.; Nakayama, T.; Inoue, E.; Sumya, N.; Mori, R. Cluster Randomised Controlled Trial Showed That Maternal and Child Health Handbook Was Effective for Child Cognitive Development in Mongolia. *Acta Paediatr. Int. J. Paediatr.* **2017**, *106*, 1360–1361, doi:10.1111/apa.13864.
38. Emond, A.; Howat, P.; Evans, J.A. Reliability of Parent-Held Child Health Records. *Health Visitor* **1995**, *68*, 322–323.
39. Hampshire, A.J.; Blair, M.E.; Crown, N.S.; Avery, A.J.; Williams, E.I. Variation in How Mothers, Health Visitors and General Practitioners Use the Personal Child Health Record. *Child. Care Health Dev.* **2004**, *30*, 307–316, doi:10.1111/j.1365-2214.2004.00433.x.
40. Harrison, D.; Heese, H.D.V.; Harker, H.; Mann, M.D. An Assessment of the "Road-to-Health" Card Eased on Perceptions of Clinic Staff and Mothers. *S. Afr. Med. J.* **1998**, *88*, 1424–1428.
41. Hikita, N.; Haruna, M.; Matsuzaki, M.; Shiraishi, M.; Takehara, K.; Dagvadorj, A.; Sumya, N.; Bavuusuren, B.; Baljinnyam, P.; Ota, E.; et al. Utilisation of Maternal and Child Health Handbook in Mongolia: A Cross-Sectional Study. *Health Educ. J.* **2018**, *77*, 458–469, doi:10.1177/0017896917753649.
42. Jeffs, D.; Nossar, V.; Bailey, F.; Smith, W.; Chey, T. Retention and Use of Personal Health Records: A Population-Based Study. *J. Paediatr. Child Health* **1994**, *30*, 248–252.
43. Jessop, L.; Lotya, J.; Murrin, C.; Fallon, U.B.; Kelleher, C.C. Relationship between Parent Held Child Records for Immunisations, Parental Recall and Health Service. *Ir. Med. J.* **2011**, *104*, 1–4.
44. Kawakatsu, Y.; Sugishita, T.; Oruenjo, K.; Wakhule, S.; Kibosia, K.; Were, E.; Honda, S. Effectiveness of and Factors Related to Possession of a Mother and Child Health Handbook: An Analysis Using Propensity Score Matching. *Health Educ. Res.* **2015**, *30*, 935–946, doi:10.1093/her/cyv048.
45. Koh, H.C.; Ang, S.K.T.; Kwok, J.; Tang, H.N.; Wong, C.M.; Daniel, L.M.; Goh, W. The Utility of Developmental Checklists in Parent-Held Health Records in Singapore. *J. Dev. Behav. Pediatr.* **2016**, *37*, 647–656, doi:10.1097/DBP.0000000000000305.
46. Lakhani, A.D.; Avery, A.; Gordon, A.; Tait, N. Evaluation of a Home Based Health Record Booklet. *Arch. Dis. Child.* **1984**, *59*, 1076–1081, doi:10.1136/adc.59.11.1076.
47. Mukanga, D.O.; Kiguli, S. Factors Affecting the Retention and Use of Child Health Cards in a Slum Community in Kampala, Uganda, 2005. *Matern. Child Health J.* **2006**, *10*, 545–552, doi:10.1007/s10995-006-0132-9.
48. Palombo, C.N.T.; Duarte, L.S.; Fujimori, E.; Toriyama, Á.T.M. Use and Records of Child Health Handbook Focused on Growth and Development. *Rev. Esc. Enferm.* **2014**, *48*, 59–66, doi:10.1590/S0080-623420140000600009.
49. Tarwa, C.; De Villiers, F. The Use of the Road to Health Card in Monitoring Child Health. *S. Afr. Fam. Pract.* **2007**, *49*, doi:10.1080/20786204.2007.10873497.
50. Troude, P.; L'Hélias, L.F.; Raison-Boulley, A.M.; Castel, C.; Bouyer, J.; De La Rochebrochard, E. Apgar Scores Reported in Personal Child Health Records: Validity for Epidemiological Studies? *J. Paediatr. Child Health* **2008**, *44*, 665–669, doi:10.1111/j.1440-1754.2008.01379.x.
51. Walton, S.; Bedford, H. Parents' Use and Views of the National Standard Personal Child Health Record: A Survey in Two Primary Care Trusts. *Child. Care Health Dev.* **2007**, *33*, 744–748, doi:10.1111/j.1365-2214.2007.00735.x.
52. Wright, C.M.; Reynolds, L. How Widely Are Personal Child Health Records Used and Are They Effective Health Education Tools? A Comparison of Two Records. *Child. Care Health Dev.* **2006**, *32*, 55–61, doi:10.1111/j.1365-2214.2006.00575.x.
53. Phillips, D.E.; Dieleman, J.L.; Lim, S.S.; Shearer, J. Determinants of effective vaccine coverage in low and middle-income countries: a systematic review and interpretive synthesis. *BMC health services research* **2017**, *17*, 681, doi:10.1186/s12913-017-2626-0.
54. Kochhar, S.; Rath, B.; Seeber, L.D.; Rundblad, G.; Khamesipour, A.; Ali, M. Introducing New Vaccines in Developing Countries. *Expert Rev. Vaccines* **2013**, *12*, 1465–1478, doi:10.1586/14760584.2013.855612.
55. Greenwood, B. The Contribution of Vaccination to Global Health: Past, Present and Future. *Philos. Trans. R. Soc. B Biol. Sci.* **2014**, *369*, 20130433, doi:10.1098/rstb.2013.0433.
56. Zewdie, A.; Letebo, M.; Mekonnen, T. Reasons for Defaulting from Childhood Immunization Program: A Qualitative Study from Hadiya Zone, Southern Ethiopia. *BMC Public Health* **2016**, *16*, 1240, doi:10.1186/s12889-016-3904-1.
57. Fawzi, W.W.; Chalmers, T.C.; Herrera, M.G.; Mosteller, F. Vitamin A Supplementation and Child Mortality. *JAMA* **1993**, *269*, 898, doi:10.1001/jama.1993.03500070078033.
58. Jones, G.; Steketee, R.W.; Black, R.E.; Bhutta, Z.A.; Morris, S.S.; Bellagio Child Survival Study Group. How Many Child Deaths Can We Prevent This Year? *Lancet* **2003**, *362*, 65–71, doi:10.1016/S0140-6736(03)13811-1.
59. Blondel, B.; Dutilhb, P.; Delourb, M.; Uzanc, S. Poor Antenatal Care and Pregnancy Outcome. *Eur. J. Obstet. Gynecol. Reprod. Biol.* **1993**, *50*, 191–196.
60. Raatikainen, K.; Heiskanen, N.; Heinonen, S. Under-Attending Free Antenatal Care Is Associated with Adverse Pregnancy Outcomes. *BMC Public Health* **2007**, *7*, 268, doi:10.1186/1471-2458-7-268.

61. Victora, C.G.; Bahl, R.; Barros, A.J.D.; França, G.V.A.; Horton, S.; Krasevec, J.; Murch, S.; Sankar, M.J.; Walker, N.; Rollins, N.C.; et al. Breastfeeding in the 21st Century: Epidemiology, Mechanisms, and Lifelong Effect. *Lancet* **2016**, *387*, 475–490, doi:10.1016/S0140-6736(15)01024-7.
62. Wu, T.; Shi, J.; Bao, S.; Qu, Y.; Mu, D.Z. Effect of Premature Rupture of Membranes on Maternal Infections and Outcome of Preterm Infants. *Zhongguo Dang Dai Er Ke Za Zhi* **2017**, *19*, 861–865.
63. Aihara, Y. *Effect of Maternal and Child Health Handbook on Maternal and Child Health Promoting Belief and Action*; Mahidol University: Nakhon Pathom, Thailand, 2005.
64. Kientz, J.A.; Arriaga, R.I.; Abowd, G.D. Baby Steps: Evaluation of a System to Support Record-Keeping for Parents of Young Children. In Proceedings of the SIGCHI Conference on Human Factors in Computing Systems, Boston, MA, USA, 4–9 April 2009; pp. 1713–1722, doi:10.1145/1518701.1518965.
65. Kitabayashi, H.; Chiang, C.; Al-Shoaibi, A.A.A.; Hirakawa, Y.; Aoyama, A. Association Between Maternal and Child Health Handbook and Quality of Antenatal Care Services in Palestine. *Matern. Child Health J.* **2017**, *21*, 2161–2168, doi:10.1007/s10995-017-2332-x.
66. Ahmadi, M.; Jeddi, F.R.; Gohari, M.R.; Sadoughi, F. A Review of the Personal Health Records in Selected Countries and Iran. *J. Med. Syst.* **2012**, *36*, 371–382, doi:10.1007/s10916-010-9482-3.
67. Brown, D.W.; Gacic-Dobo, M. Home-Based Record Prevalence among Children Aged 12–23 Months from 180 Demographic and Health Surveys. *Vaccine* **2015**, *33*, 2584–2593, doi:10.1016/j.vaccine.2015.03.101.
68. Newacheck, P.W.; Halfon, N. The Financial Burden of Medical Care Expenses for Children. *Med. Care* **1986**, *24*, 1110–1117.
69. Moss, A.L.H. Is the Personal Child Health Record Used in Secondary Care? *Child. Care Health Dev.* **2005**, *31*, 627–628, doi:10.1111/j.1365-2214.2005.00533.x.
70. Osaki, K.; Hattori, T.; Kosen, S.; Singgih, B. Investment in Home-Based Maternal, Newborn and Child Health Records Improves Immunization Coverage in Indonesia. *Trans. R. Soc. Trop. Med. Hyg.* **2009**, *103*, 846–848, doi:10.1016/j.trstmh.2009.03.011.
71. Osaki, K.; Hattori, T.; Kosen, S. The Role of Home-Based Records in the Establishment of a Continuum of Care for Mothers, Newborns, and Children in Indonesia. *Glob. Health Action* **2013**, *6*, doi:10.3402/gha.v6i0.20429.
72. Osaki, K.; Kosen, S.; Indriasih, E.; Pritasari, K.; Hattori, T. Factors Affecting the Utilisation of Maternal, Newborn, and Child Health Services in Indonesia: The Role of the Maternal and Child Health Handbook. *Public Health* **2015**, *129*, 582–586, doi:10.1016/j.puhe.2015.01.001.
73. Tom, J.O.; Chen, C.; Zhou, Y.Y. Personal Health Record Use and Association with Immunizations and Well-Child Care Visits Recommendations. *J. Pediatr.* **2014**, *164*, 112–117, doi:10.1016/j.jpeds.2013.08.046.
74. Mahomed, K.; Mason, E.; Warndorf, T. Home-Based Mother's Record: Operational Feasibility, Understanding and Usage in a Rural Community in Zimbabwe. *Trop. Doctor* **2000**, *30*, 155–159, doi:10.1177/004947550003000314.
75. Kelly, R.G. Midwifery and Child Health Nursing: Supporting Early Parenting Mental Wellbeing. Ph.D. Thesis, University of Tasmania, Hobart, Australia 2014.
76. Whetstone, M.; Goldsmith, R. Factors Influencing Intention to Use Personal Health Records. *Int. J. Pharm. Healthc. Mark.* **2009**, *3*, 8–25, doi:10.1108/17506120910948485.
77. Carr, V.J.; Harris, F.; Raudino, A.; Luo, L.; Kariuki, M.; Liu, E.; Tzoumakis, S.; Smith, M.; Holbrook, A.; Bore, M.; et al. New South Wales Child Development Study (NSW-CDS): An Australian Multiagency, Multigenerational, Longitudinal Record Linkage Study. *BMJ Open* **2016**, *6*, doi:10.1136/bmjopen-2015-009023.
78. Eapen, V.; Woolfenden, S.; Williams, K.; Jalaludin, B.; Dissanayake, C.; Axelsson, E.L.; Murphy, E.; Eastwood, J.; Descallar, J.; Beasley, D.; et al. Are You Available for the next 18 Months? A Longitudinal Birth Cohort Study Investigating a Universal Developmental Surveillance Program: The 'Watch Me Grow' Study. *BMC Pediatr.* **2014**, *14*, 234, doi:10.1186/1471-2431-14-234.
79. Froen, J.F.; Myhre, S.L.; Frost, M.J.; Chou, D.; Mehl, G.; Say, L.; Cheng, S.; Fjeldheim, I.; Friberg, I.K.; French, S.; et al. ERegistries: Electronic Registries for Maternal and Child Health. *BMC Pregnancy Childbirth* **2016**, *16*, 11, doi:10.1186/s12884-016-0801-7.
80. Knowles, R.; Blackburn, M.; Zahir, M.; Russell, M.; Carrier, A.; Nevrkla, E. The Implementation of a New Parallel Child Health Record. *Child Care Health Dev.* **1999**, *25*, 253–266.
81. Thomas, R.E.; Spragins, W.; Mazloum, G.; Cronkhite, M.; Maru, G. Rates of Detection of Developmental Problems at the 18-Month Well-Baby Visit by Family Physicians' Using Four Evidence-Based Screening Tools Compared to Usual Care: A Randomized Controlled Trial. *Child. Care Health Dev.* **2016**, *42*, 382–393, doi:10.1111/cch.12333.
82. Knight, A.W.; Szucs, C.; Dhillon, M.; Lembke, T.; Mitchell, C. The ECollaborative: Using a Quality Improvement Collaborative to Implement the National Ehealth Record System in Australian Primary Care Practices. *Int. J. Qual. Health Care* **2014**, *26*, 411–417, doi:10.1093/intqhc/mzu059.
83. Kim, E.H.; Modi, S.; Fang, D.; Soh, C.B.; Herbaugh, A.; Shinstrom, S.; Lober, W.B.; Zierler, B.; Kim, Y. Web-Based Personal-Centered Electronic Health Record for Elderly Population. In Proceedings of the 1st Transdisciplinary Conference on Distributed Diagnosis and Home Healthcare, Arlington, VA, USA, 2–4 April 2006; 144–147, doi:10.1109/DDHH.2006.1624817.

84. Kim, M.I.; Johnson, K.B. Personal Health Records: Evaluation of Functionality and Utilty. *J. Am. Med. Inform. Assoc.* **2002**, *9*, 171–180.
85. Reich, S.M.; Penner, E.K.; Duncan, G.J. Using Baby Books to Increase New Mothers' Safety Practices. *Acad. Pediatr.* **2011**, *11*, 34–43, doi:10.1016/J.ACAP.2010.12.006.
86. Rybynok, V.O.; Kyriacou, P.A.; Binnersley, J.; Woodcock, A. MyCare Card Development: Portable GUI Framework for the Personal Electronic Health Record Device. *IEEE Trans. Inf. Technol. Biomed.* **2011**, *15*, 66–73, doi:10.1109/TITB.2010.2091143.
87. White, D. Using a Patient Held Record for Home Based Palliative Care Patients : A Case Study Research Project. Master's Thesis, Massey University, Manawatu, New Zealand, 2012.
88. Nurhayati, S.; Sistiarani, C.; Dardjito, E. Descriptive study quality improvement of using mch book. *Kesmasindo* **2014**, *7*, 54–63.
89. Wenzel, V. *Use of Electronic Health Records to Aid in Pediatric Obesity Diagnosis*; Cornell University: Ithaca, NY, USA, 2015.

© 2019 by the authors. Submitted for possible open access publication under the terms and conditions of the Creative Commons Attribution (CC BY) license (http://creativecommons.org/licenses/by/4.0/).
